# Implementation and Educational Impact of a Story-Centered Curriculum Using a Large Language Model: A Class on Internal Disorders for Physiotherapy Students

**DOI:** 10.7759/cureus.94390

**Published:** 2025-10-12

**Authors:** Shota Okuno, Kenta Kawamitsu, Tamotsu Yamaguchi

**Affiliations:** 1 Department of Rehabilitation, Aso Iizuka Hospital, IIzuka, JPN; 2 Department of Physical Therapy, Aso Rehabilitation College, Fukuoka, JPN

**Keywords:** chatgpt, large language model, physiotherapy students, simulation-based learning (sbl), story-centered curriculum

## Abstract

Background

Story-centered curricula (SCC) can effectively enhance clinical reasoning and learner engagement; however, developing high-fidelity scenarios is resource-intensive. We developed an SCC using a large language model (LLM; ChatGPT, GPT-4) to generate longitudinal respiratory cases for physiotherapy students and evaluated its educational impact.

Methodology

This single-institution, quasi-experimental study used a non-randomized historical control design. Physiotherapy students in a 15-session course were divided into an SCC group, which completed eight LLM-generated narrative sessions, and a control group, which received traditional case-based sessions without a continuous storyline. The primary outcome was the change in score on a 30-item, 300-point proficiency test administered before and after the intervention. Secondary outcomes included five Likert-scale items evaluating learner experience. Group comparisons used appropriate parametric or non-parametric tests, and multivariable linear regression adjusted for age, sex, program, and pre-test score.

Results

The final sample consisted of 169 participants, with 92 in the SCC group and 77 in the control group. Baseline characteristics and pre-test median scores were 90 with an interquartile range (IQR) of 70 to 100 in the SCC group and 90 with an IQR of 70 to 110 in the control group, showing no significant differences between groups. The SCC group demonstrated a greater improvement in test performance, with a median change of 105 and an IQR of 78 to 140, compared with a median change of 80 and an IQR of 50 to 110 in the control group (p < 0.001). The SCC group also achieved higher post-test scores, with a median of 195 and an IQR of 160 to 223, compared with a median of 180 and an IQR of 150 to 200 in the control group (p = 0.013). Positive questionnaire responses (scores of 4 or 5) exceeded 90% across all domains, including immersion 87 (94.6%) and learning retention 88 (95.7%). Participation in the SCC program remained an independent predictor of post-test performance, with a regression coefficient of 23.87 and a 95% confidence interval of 11.32 to 36.42 (p < 0.001).

Conclusions

An SCC utilizing an LLM is an innovative educational approach that effectively balances improved learning outcomes with efficient scenario development, offering significant potential to advance simulation-based education in physiotherapy.

## Introduction

In professional healthcare and rehabilitation training, simulation-based education in settings that approximate real clinical environments has proven effective [1-3]. Particularly, learning frameworks that emphasize narrative and context, where learners tackle a series of challenges as characters in a story, are crucial for integrating knowledge application with clinical reasoning. A prominent design framework for this is goal-based scenarios (GBS), which encourages “learning-by-doing,” as learners work toward achieving specific goals [4]. The story-centered curriculum (SCC) is an extension of the GBS design philosophy to encompass an entire curriculum. In an SCC, multiple GBS (goal-oriented tasks) are linked within a single, coherent narrative, allowing learners to iteratively engage in decision-making, practice, feedback, and reflection in a manner that mirrors real professional workflow [5]. Such a narrative-centered learning environment enhances learner agency, problem-solving skills, and engagement [6]. The narrative-based design is vital for integrating knowledge application and clinical reasoning within authentic professional and clinical contexts.

Furthermore, a narrative-based simulation education is expected to enhance learning outcomes by leveraging technology. A meta-analysis of technology-enhanced simulation education demonstrated positive effects on knowledge, skills, behaviors, and patient outcomes [2]. Among physiotherapists, simulation-based education has been shown to improve clinical decision-making for low back pain [7] and maintain comparable levels of clinical competence, even when replacing 25% of clinical practice hours in the cardiopulmonary field with simulation [8]. However, designing and implementing immersive scenarios that promote active learner engagement requires substantial human and time resources [9], and the high development burden, particularly the significant time commitment required from faculty, remains a major challenge to the widespread adoption of approaches such as SCC [10]. While Japan is advancing active, competency-based learning in healthcare education, constraints on faculty time and development budgets persist; at the same time, educational digitalization across the school system, including tablet-based learning in primary and secondary schools, highlights the need for scalable, technology-enabled curricula.

Large language models (LLMs) are artificial intelligence (AI) technologies trained on massive text datasets, capable of performing highly accurate tasks, such as generating text, summarizing information, and answering questions. ChatGPT (OpenAI), a rapidly evolving LLM, has demonstrated medical knowledge and explanatory capabilities comparable to passing-level performance on the United States Medical Licensing Examination [11]. Its advanced language generation and contextual retention make it a promising tool for generating cross-disciplinary narratives and clinical case scenarios [12]. Furthermore, simulation-based education using ChatGPT has provided a more realistic and immersive learning experience than traditional static case problems, as scenarios dynamically evolve in response to learner inputs [13,14]. However, current ChatGPT-based simulation education does not integrate the entire curriculum into a unified narrative, as seen in approaches such as SCC. Hence, leveraging LLMs to generate SCC narratives has the potential to minimize the development burden while maximizing the effectiveness of simulation-based education.

This study aimed to develop and evaluate an SCC utilizing ChatGPT for physiotherapy students in the field of internal disorders. The study sought to examine whether this AI-supported narrative learning approach could improve learners’ comprehension, immersion, and clinical reasoning compared with traditional case-based instruction.

## Materials and methods

Study design

This quasi-experimental study employed a non-randomized controlled trial design. The participants were physiotherapy students enrolled in a course on internal disorders at a single institution. The study included two cohorts, namely, the SCC group, comprising students from the 2025 academic year who received an intervention based on the SCC, and the control group, comprising students from the 2024 academic year who received conventional instruction. The exclusion criteria were as follows: (1) failure to provide informed consent, (2) failure to complete both pre- and post-course proficiency tests, and (3) withdrawal from the program during the study period, including leave of absence or dropout. No formal sample size calculation was performed because the aim was to evaluate the educational effectiveness of the SCC intervention across student populations.

Outcome measures

The outcome measures included age, sex, program type (daytime/evening division), and pre- and post-course proficiency test scores for both the SCC and control groups. The proficiency test consisted of 30 multiple-choice questions based on past and similar items from the Japanese national physiotherapy licensing examination. Each question was worth 10 points, with a possible maximum score of 300. The content, difficulty, and timing of the pre- and post-tests were identical for both groups, and no feedback was provided to the participants.

Additionally, participants in the SCC group completed a learner survey after the course. The survey comprised five items, rated on a five-point Likert scale, with 5 = strongly agree, 4 = agree, 3 = neutral, 2 = disagree, and 1 = strongly disagree. The five items evaluated the following domains: Understanding: the degree to which the stories helped the students develop a concrete understanding of chronic obstructive pulmonary disease (COPD) and sputum management; clinical reasoning: the extent to which the stories fostered an understanding of the rationale for specific evaluations and interventions; immersion: the degree to which the stories created a sense of presence, as if the students were physiotherapists interacting with patients; learning retention: the perceived memorability and ease of learning from the story-based format; and satisfaction: students’ overall satisfaction and desire to participate in similar courses in the future.

Course structure and educational intervention

The course on internal disorders consisted of 15 sessions, each lasting 180 minutes. The first seven sessions (sessions 1-7) focused on lectures covering the foundational knowledge of respiratory diseases, including pathophysiology, treatment, and rehabilitation. The educational intervention was implemented during the latter eight sessions (sessions 8-15). In these sessions, the SCC group participated in SCC format learning, which featured a continuous narrative centered on two fictional patient cases (Mr. T and Ms. M) generated using ChatGPT (GPT-4; OpenAI, San Francisco, CA, USA). Clinical problems related to COPD, sputum management, and physical assessment were integrated into the storyline, and students engaged in reasoning and making decisions as active participants within the narrative. In contrast, the control group participated in a problem-based learning, where a standalone case was introduced in each session. Unlike the SCC format, the control group did not receive a continuous storyline or detailed patient information. All sessions were conducted face-to-face, and online video materials were assigned as homework for preparation and review. The proficiency test was administered via Google Forms with automated scoring, ensuring consistency and minimizing evaluator bias.

Instructional material development

The instructional materials were developed based on the principles of SCC, an educational framework designed to foster practical knowledge and clinical reasoning skills by engaging learners as active participants in a narrative. The story focused on two fictional patients with respiratory diseases: Mr. T (COPD) and Ms. M (pneumonia). The narrative was generated through ChatGPT. Prompts were carefully designed to include essential clinical information, such as patient history, symptoms, physical assessment findings, medical staff comments, patient and family perspectives, and value-based decision-making. Multiple narrative variations were generated, and the final scenarios were reviewed and refined by faculty members experienced in respiratory rehabilitation to ensure clinical accuracy and educational validity. A learning management system (Google Classroom; Google LLC, Mountain View, CA, USA) was used to manage all course-related activities, including story delivery, assignment distribution, submissions, and feedback.

Example prompts used for generating the narratives are presented below.

Prompt for Mr. T (COPD): A 68-year-old male with COPD (GOLD stage III, receiving home oxygen therapy) presents complaints of sputum retention and exertional dyspnea. Provide clinical information, including age, medical history, vital signs, respiratory sounds, walking test results, and other relevant findings, in chronological order. Include comments from physicians and nurses, patient statements, and family perspectives to create a realistic and immersive narrative suitable for student learning.

Prompt for Ms. M (Pneumonia): A 78-year-old female hospitalized with pneumonia, with a history of diabetes and hypertension. On admission, she had a fever of 38°C and a productive cough, with chest X-ray showing infiltrates in the right lower lung field and elevated inflammatory markers. Nursing notes indicate reduced appetite, and the family expressed concerns about her ability to return to living independently after discharge. Create a scenario where students act as physiotherapists conducting the initial evaluation.

To enhance learning engagement, Notebook LM (formerly Project Tailwind; Google LLC) was used to generate audio narration scripts based on the story texts, allowing students to experience the material visually and auditorily. The complete story scripts are provided in the Appendices.

Educational effect evaluation

The educational effects were evaluated using two measures, i.e., a learner questionnaire and a proficiency test. The learner questionnaire assessed the following five domains: understanding, clinical reasoning, immersion, learning retention, and satisfaction. The primary outcome was the change in the proficiency test score (Δ Score), calculated as the difference between the final and initial test scores.

Statistical analysis

The responses from the learner questionnaire were summarized for each domain in the SCC group. Subsequently, comparisons between the SCC and control groups were conducted. Statistical tests were selected according to the data type and distribution. Continuous variables were expressed as medians with interquartile ranges (IQRs), regardless of normality. Between-group comparisons used the t-test for normally distributed variables, the Wilcoxon rank-sum test for non-normally distributed variables, and the chi-square test for categorical variables. To identify independent factors associated with knowledge improvement, a multiple linear regression analysis was performed. The change in proficiency test score (Δ Score) was the dependent variable, and SCC participation, age, sex, program type (daytime/evening division), and pre-course proficiency test score were the independent variables. This analysis allowed the evaluation of the independent effect of SCC while adjusting for baseline characteristics. Regression coefficients, 95% confidence intervals (CIs), and p-values were reported. All analyses were performed using R statistical software (version 4.4.1), and a p-value <0.05 was considered statistically significant.

## Results

**Figure 1:**
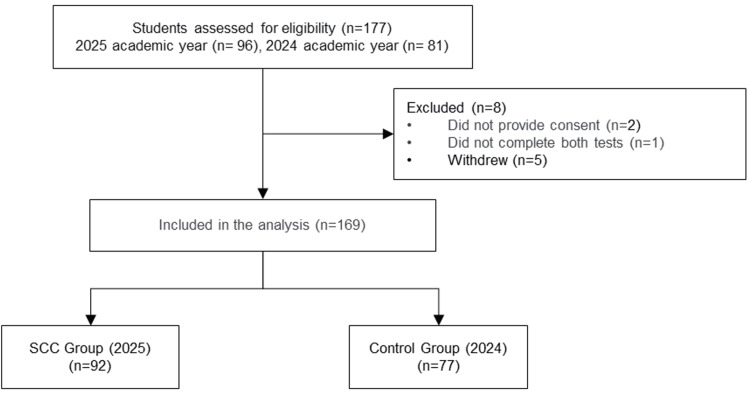
Flowchart of student inclusion and exclusion. SCC: story-centered curriculum

The final sample consisted of 169 participants, with 92 in the SCC group and 77 in the control group (Figure 1). The median age was 19.5 years for the SCC group and 20.0 years for the control group. The SCC group included 31 (34%) males, compared with 30 (39%) males in the control group. There were no statistically significant differences between the SCC and control groups concerning age, sex, program type, or other baseline characteristics. Detailed participant characteristics are presented in Table 1.

**Table 1:** Participant characteristics in the control and SCC groups. Note: values are presented as n (%) or median (IQR). Between-group comparisons were performed using the t-test for normally distributed continuous variables, the Wilcoxon rank-sum test for non-normally distributed variables, and the chi-square test for categorical variables. Test statistics are reported as χ² for chi-square tests, W for Wilcoxon rank-sum tests, and U for Mann–Whitney U tests. SCC: story-centered curriculum; Δ Score: difference between post- and pre-test scores; IQR: interquartile range

Characteristic	SCC group (N = 92)	Control group (N = 77)	Test statistic	P-value
Age, year	19.5 (19.0, 21.0)	20.0 (19.0, 20.0)	U = 3,781	0.507
Sex				
Male	31 (34)	30 (39)	χ² = 0.51	0.583
Female	61 (66)	47 (61)		
Program				
Daytime	75 (82)	65 (84)	χ² = 0.16	0.770
Evening	17 (18)	12 (16)		
Pre-test score, points	90 (70, 100)	90 (70, 110)	W = 4,228	0.085
Post-test score, points	195 (160, 223)	180 (150, 200)	W = 2,841	0.013
Δ Score, points	105 (78, 140)	80 (50, 110)	W = 2,403	<0.001

Learner questionnaire outcomes

All 92 participants in the SCC group completed the post-course learner questionnaire, resulting in a 100% response rate. Positive responses, defined as scores of 4 or 5, exceeded 90% for all five evaluated domains: understanding 85 (92.4%), clinical reasoning 89 (96.7%), immersion 87 (94.6%), learning retention 88 (95.7%), and satisfaction 85 (92.4%) (Table 2).

**Table 2:** Results of the post-class questionnaire. Note: data are presented as n (%), representing the number of respondents and the corresponding percentage within each response category. Positive responses were defined as the combined proportion of scores 4 and 5.

Item	5 (strongly agree)	4 (agree)	3 (neutral)	2 (disagree)	1 (strongly disagree)	Positive responses (4+5, %)
Understanding	41 (44.6)	44 (47.8)	4 (4.3)	2 (2.2)	1 (1.1)	85 (92.4)
Clinical reasoning	46 (50.0)	43 (46.7)	3 (3.3)	0 (0.0)	0 (0.0)	89 (96.7)
Immersion	58 (63.0)	29 (31.5)	4 (4.3)	0 (0.0)	1 (1.1)	87 (94.6)
Learning retention	57 (62.0)	31 (33.7)	4 (4.3)	0 (0.0)	0 (0.0)	88 (95.7)
Satisfaction	60 (65.2)	25 (27.2)	6 (6.5)	1 (1.1)	0 (0.0)	85 (92.4)

Proficiency test outcomes

There was no significant difference in median pre-course proficiency test scores between the SCC group (90 (IQR 70 to 100)) and the control group (90 (70 to 110)). However, post-course median scores were significantly higher in the SCC group compared to the control group (195 (160 to 223) vs. 180 (150 to 200); p = 0.013). Furthermore, the median change in proficiency test scores was greater in the SCC group (105 (78 to 140)) compared to the control group (80 (50 to 110); p < 0.001) (Table 1).

Multiple regression analysis

Multiple linear regression demonstrated that participation in the SCC intervention was an independent predictor of greater improvement in proficiency test scores (β = 23.87, 95% CI 11.32 to 36.42; p < 0.001), even after adjusting for age, sex, program type, and pre-course proficiency test scores (Table 3).

**Table 3:** Results of the multiple regression analysis for the change in proficiency test scores. SCC: story-centered curriculum; β: unstandardized regression coefficient; CI: confidence interval

Variable	β	95% CI	P-value
SCC group	23.87	11.32, 36.42	<0.001
Age (years)	0.63	-1.00, 2.26	0.448
Sex (male)	-11.70	-24.71, 1.30	0.077
Pre-test score	-0.33	-0.55, -0.10	0.005
Program (evening)	2.50	-15.13, 20.13	0.780

## Discussion

This study found that participation in an SCC incorporating an LLM was associated with two key outcomes: (1) enhanced learner satisfaction and immersion, and (2) improved comprehension, knowledge retention, and performance on proficiency tests.

The learner questionnaire results indicated that the LLM-based SCC developed in this study successfully promoted satisfaction and immersion. This is consistent with previous findings, whereby case-based and story-driven learning approaches have been reported to enhance learner satisfaction [15]. As a theoretical basis for the role of immersion in promoting learning, Dede [16] proposed that immersive interfaces, such as virtual reality (VR) and augmented reality (AR), enabled situated learning by allowing learners to acquire knowledge within authentic contexts and activities. Furthermore, learning in contexts that closely resemble real-world situations facilitated the transfer of acquired knowledge to practical settings.

However, systematic reviews have noted that implementing and maintaining immersive interfaces, such as VR technology, involves substantial initial costs [17]. Particularly, within healthcare education, the high initial investment and ongoing maintenance of equipment remain major barriers [18]. In addressing these challenges, the text-based LLM approach adopted in this study offers the advantage of enabling the design of a relatively low-cost virtual environment where learners can practice history-taking, clinical reasoning, and communication skills through simulated patient interactions [19]. This study has practical significance in applying the advantages of LLMs within the SCC framework, successfully achieving high immersion and educational effectiveness in a cost-efficient manner.

The LLM-based SCC developed in this study improved comprehension, clinical reasoning, and knowledge retention, resulting in higher proficiency test scores. The finding that SCC enhanced learners’ subjective evaluations and objective test performance can be interpreted from the perspective of cognitive learning mechanisms. This improvement aligns strongly with situated learning theory [20], which posits that learning is most effective when it occurs within authentic, practice-based contexts. In medical education, case-based learning has proven to enhance knowledge organization and clinical application by providing contextual frameworks, as demonstrated in a systematic review by Thistlethwaite [21]. The SCC developed in this study builds upon this principle by offering a richer context through a continuous narrative, promoting deeper knowledge application and retention.

Furthermore, the principle of retrieval practice may have contributed to the learning gains. Repeatedly retrieving information from memory has consistently been demonstrated in cognitive psychology research to enhance long-term knowledge retention [22]. Within the SCC in this study, learners were repeatedly required to evaluate changing patient conditions and make intervention decisions throughout the storyline, which integrated the retrieval and application of knowledge into the learning process. This repeated engagement likely reinforced memory consolidation and contributed to improved proficiency test scores.

Nonetheless, certain limitations of this study must be acknowledged. This was a single-institution, non-randomized historical control study and, therefore, is susceptible to confounding factors, such as cohort differences, student background, faculty experience, and influences outside the curriculum. However, even after controlling for potential confounders through multiple regression analysis, significant differences in Δ proficiency test scores remained, which is noteworthy. The primary outcomes were limited to short-term knowledge acquisition. Long-term retention, clinical performance, and patient-related outcomes were not assessed, indicating the need for longitudinal studies.

Despite these limitations, this study makes significant contributions. It demonstrates that educators can leverage LLMs as “creative partners” to efficiently develop high-quality, context-rich learning materials. This can accelerate the implementation and dissemination of simulation-based education, particularly in resource-limited settings. Future directions include harnessing LLMs interactively to create adaptive learning environments in which storylines dynamically branch based on learners’ responses, providing personalized and optimized educational experiences.

## Conclusions

This study demonstrated that the SCC significantly improved participants’ test performance, learning retention, and immersion compared to conventional approaches. These findings suggest that integrating SCC into clinical education provides a more engaging and effective training experience, fostering active participation and sustained motivation. The consistently positive feedback from learners indicates that SCC is both feasible and acceptable, underscoring its potential for broader implementation in physiotherapy and other health professional education. By confirming SCC’s effectiveness, our results offer valuable guidance for educators seeking innovative approaches to enhance clinical education and point toward a promising direction for future curriculum development.

## Appendices

Case T teaching materials (English)

**Table 4:** Student-facing case script and assignments (Case T). This table reproduces selected portions of the narrative script, stakeholder dialogues, and written assignments as delivered to students during Sessions 8-15 of the SCC course. COPD: chronic obstructive pulmonary disease; HOT: home oxygen therapy; 5STS: five-times sit-to-stand; 6MWD: six-minute walk distance; HADS: Hospital Anxiety and Depression Scale; FIM: Functional Independence Measure; NRADL: Nagasaki University Respiratory ADL Questionnaire

Case script and student tasks	
S1. Case narrative	
Mr. T is 68 years old and lives with his wife. Their adult daughter lives in Oita and they see each other about once a year. Three years ago, his primary physician diagnosed him with COPD. He has a long smoking history and had assumed that cough and shortness of breath were just “signs of aging,” but over the past few years his dyspnea has clearly worsened. Last year he was hospitalized twice for acute exacerbations of COPD. HOT was initiated thereafter. He uses 1 L/minute at rest and 2 L/minute during activity. In addition to pharmacotherapy including tiotropium (Spiriva), he is also being treated for comorbid benign prostatic hyperplasia, hypertension, and anxiety. He takes etizolam at night and reports that he “falls asleep easily.” On recent assessment, his BMI was 17.4 kg/m² (underweight), skeletal muscle mass index (SMI) 6.2 kg/m², grip strength 22–24 kg, five-times sit-to-stand time 22 seconds, and gait speed relatively preserved at 1.20 m/s. However, his 6MWD was 300 m and he required two rests. He states, “I get short of breath quickly when I walk. The bathroom is especially tough.” His GOLD classification is stage III (severe), group D. His MRC dyspnea scale is grade 4. Both sarcopenia and frailty are present. Hospital Anxiety and Depression Scale (HADS) scores are 8 for anxiety and 13 for depression, indicating a prominent depressive tendency. Despite this, his Functional Independence Measure (FIM) score is 126 (full score). He is independent in daily life and reports not relying on his wife for assistance. Nonetheless, he says, “I can only manage a bath once a week.” He has become reluctant to use stairs or go out, saying, “It’s scary and exhausting.” His Nottingham Extended Activities of Daily Living (NRADL) score is 68. During the initial interview he said: “I’d rather spend my time quietly at home. I’ve told my daughter not to push herself to come. Even with rehabilitation, this isn’t a disease that can be cured…”	
S2. Perspectives from professionals, family members, and the patient	
Stakeholder (speaker)	Dialogue (verbatim)
Physician’s perspective	Mr. T has experienced recurrent COPD exacerbations this year. We have adjusted his inhaled medications, and I would like to request inpatient rehabilitation during this admission. Although the patient himself is not very motivated for rehabilitation, his wife and daughter strongly hope to prevent any further deconditioning. There are no activity restrictions. Thank you for your assistance.
Nurse’s perspective	“Mr. T used to be an active person, but since starting home oxygen therapy, he has become more homebound. He often complains of nighttime shortness of breath and anxiety, and his sleep quality does not seem to be very good. In addition, his wife is very concerned and is unsure how much support she should provide in daily life. Although Mr. T does not seem very motivated to participate in rehabilitation, he does appear to be worried about his declining physical strength.”
Family’s perspective	He used to be an active person and enjoyed going out for walks. Since starting oxygen therapy, he has spent most of his time at home. His expression has become subdued, and he has been eating less. Can rehabilitation help in any way?
Patient’s voice	“To be honest, I feel there’s no point in doing rehabilitation. Of course, if this breathlessness went away, I’d love to take a bath every day and go for walks outside… (pauses) Actually, I’d really like to visit my grandchild who lives in Oita, but… since this disease can’t be cured, I guess there’s nothing I can do.”
S3. Assignments	
Session 1 written assignment	Read the following story and describe what you could do for Mr. T based on the knowledge you have acquired so far as a student. You are still a “physical therapist in training.” You are unsure about even basic things such as how to read charts or how to speak with patients. Today you are assigned to Mr. T, a 78‑year‑old man with COPD. His chart states “Initial PT evaluation not yet performed.” When you enter his room, he is watching TV. When you greet him, he glances at you and shakes his head slightly. “No, I’m fine. Doing rehab now won’t change anything.” A brief silence follows. You muster the courage to say, “But… I heard bathing and walking have been difficult. Is there anything you would actually like to be able to do?” He averts his eyes and murmurs, “…To be honest, I’d like to bathe every day and go out for a walk. I’d like to visit my grandchild in Oita, too… but it’s impossible now.” You are at a loss for words. You want to believe there is something you can do, but you also feel anxious about your own lack of experience. This is today’s final assignment. High‑quality submissions will receive +2 bonus points and may be shared in the next class. Sample dialogue: Mr. T: “I can manage my daily life on my own. There’s no point in doing rehab.” You: “But I heard you feel short of breath in daily life and cannot go out, right?” Mr. T: “The doctor told me COPD doesn’t get cured. I’m fine staying at home.” You: “Is there something you would really like to be able to do?” Mr. T: “If this breathlessness improved, I’d like to bathe every day and take a walk… (silence) Honestly, I’d like to visit my grandchild in Oita, but… it’s a disease that won’t be cured, so it can’t be helped.” Assignment: Mr. T appears to think physical therapy may not be meaningful. As a physical therapy student, what could you do for him? Explain the potential benefits of physical therapy for Mr. T and what specific actions would be needed to achieve them. *Note: Consider the role of PTs as movement specialists.
Session 2 written assignment	Building on the previous session, what can you do for Mr. T now? What could you propose to reduce dyspnea in daily life? This is your second time working with Mr. T, 78, who initially said “No thanks—rehab won’t help me.” Today feels a bit different. After a light exercise in the PT gym, he smiles faintly and says, “When I exercised, the breathlessness wasn’t as bad as I expected… I was a little happy.” You nod. Trying to move forward step by step, he says, “But getting dressed and bathing are still tough.” You think to yourself: “Why does he get so breathless after moving? Why when raising his arms or bending forward?” You decide to revisit his condition and symptoms. Using key concepts such as “dynamic hyperinflation” and “accessory respiratory muscles,” explain the mechanisms linking COPD pathology to symptoms. This is today’s final assignment. High‑quality submissions will receive +1 bonus point and may be shared in the next class. Sample dialogue: Mr. T: “I was happy to find that exercise didn’t make me as breathless as I thought.” You: “That’s great. How about daily life?” Mr. T: “Getting dressed and taking a bath are still hard.” You: “I see.” Assignment: Mr. T has become a bit more positive toward PT. What can you do so that he can dress and bathe with less breathlessness? Describe both immediate strategies (short‑term relief) and longer‑term approaches. *Note: Consider the PT’s expertise in movement analysis.*
Session 3 written assignment	Mr. T does not seem to be gaining much muscle strength. Based on the following conversation, consider specific strategies to help Mr. T increase both his body weight and muscle strength. Conversation: As Mr. T was leaving the rehabilitation session, he suddenly spoke up: “You know, I’ve actually been working pretty hard. I’ve been doing the exercises you taught me every day. But… somehow, my strength hasn’t improved much.” Surprised, you checked his chart. Indeed, the measurements for grip strength and lower-limb muscle strength haven’t changed significantly. Then your eyes caught something in the weight section. “Mr. T, I see you’ve lost a little weight recently.” “Yeah, that’s right. I haven’t had much of an appetite lately. In the morning I just eat a slice of bread, and sometimes I just have jelly for lunch. But since I’m still moving around, I figured it was fine.” Assignment: As a physical therapy student, propose concrete strategies to help Mr. T improve both his muscle strength and body weight. Make sure to consider aspects such as: Exercise prescription (type, intensity, and frequency) Nutritional support (collaboration with dietitians, protein intake, meal planning) Daily activity planning (balancing rest and activity to prevent fatigue). Note: This English file includes the core narrative and the first three session assignments for Case T. Additional sessions and figures can be appended in the same format.
